# Teratozoospermia and Embryo Development: The Significance of Sperm Selection in In Vitro Fertilization Success

**DOI:** 10.3390/jcm14113763

**Published:** 2025-05-27

**Authors:** Petronela Naghi, Ioana Alexandra Zaha, Liana Stefan, Andrea Sorian, Adelin Marcu, Liliana Sachelarie, Anca Huniadi

**Affiliations:** 1 Calla—Infertility Diagnostic and Treatment Center, Constantin A. Rosetti Street, 410103 Oradea, Romania; petronelanaghi@gmail.com (P.N.); izaha@uoradea.ro (I.A.Z.); sorian.andrea@gmail.com (A.S.); adelinmarcu890@yahoo.com (A.M.); ahuniadi@uoradea.ro (A.H.); 2 Faculty of Medicine and Pharmacy, University of Oradea, 1st December Square 10, 410073 Oradea, Romania; 3 Pelican Clinical Hospital, Corneliu Coposu Street 2, 410450 Oradea, Romania; 4 Oradea County Hospital, Gheorghe Doja Street 65–67, 410169 Oradea, Romania; 5 Department of Clinical Discipline, Apollonia University, 700511 Iasi, Romania

**Keywords:** teratozoospermia, sperm morphology, sperm selection, blastocyst development, assisted reproductive technology, microfluidic sperm sorting

## Abstract

**Background:** Sperm morphology is a key factor influencing fertilization and embryo development in assisted reproductive technology (ART). However, the predictive value of sperm deformity indices and selection techniques remains debated. This study evaluated the impact of teratozoospermia on fertilization, blastocyst formation, and embryo quality, comparing conventional and microfluidic sperm selection methods. **Methods:** A retrospective analysis was conducted on ART cycles involving patients with teratozoospermia. Sperm selection was performed using density gradient centrifugation (DGC) or microfluidic sperm sorting (MFSS). The correlations between the Sperm Deformity Index (SDI), Multiple Anomalies Index (MAI), and Teratozoopermia Index (TZI) with fertilization rates, blastocyst formation, and embryo quality were assessed. Statistical analysis included correlation tests, receiver operating characteristic (ROC) curves, and independent samples *t*-tests. **Results:** Patients with severe teratozoospermia exhibited lower fertilization rates (*p* < 0.01) and reduced blastocyst formation (*p* = 0.02). The SDI and MAI showed moderate negative correlations with fertilization (r = −0.15 and r = −0.25, respectively) and blastocyst development (r = −0.20 and r = −0.30, respectively), while the TZI had only weak associations (r = −0.10 and r = −0.15, respectively). ROC analysis demonstrated that the SDI and MAI were moderate predictors of embryo viability (AUC = 0.70 and 0.75, respectively). Patients who underwent microfluidic sperm selection had higher fertilization rates (*p* = 0.03) and improved blastocyst quality (*p* = 0.04) than those processed with DGC. **Conclusions:** Severe teratozoospermia negatively affects fertilization and blastocyst formation, with the SDI and MAI showing moderate predictive value for embryo development. The use of microfluidic sperm selection significantly improved embryo quality, supporting its clinical relevance in ART.

## 1. Introduction

The quality of embryos for implantation depends on the functional, morphological, and structural integrity of male gametes (sperm) and female gametes (oocytes). Evaluating the male partner’s contribution to embryos produced via IVF is complex. While male infertility is often linked to identifiable causes, over 50% of cases are unexplained or idiopathic [1].

Sperm from infertile men may carry structural, functional, chromosomal, or genetic abnormalities. These can occur even in the presence of normal or slightly altered semen parameters. For managing subfertility in men, semen analysis remains the cornerstone of diagnosis, encompassing evaluations of morphology, motility, dynamics, and functional characteristics [1,2].

Infertility is a multifactorial condition with causes classified into female, male, combined, and unexplained factors. Among these, unexplained infertility is frequently considered the reference category when comparing IVF success rates. Studies indicate that pregnancy outcomes tend to be more favorable in unexplained infertility than in cases of severe male factor infertility, where sperm abnormalities directly impact fertilization and embryo development. Since sperm morphology plays a crucial role in reproductive success, this study explicitly evaluated the impact of teratozoospermia on blastocyst formation and implantation potential. By analyzing the predictive value of sperm deformity indices and selection techniques, we aimed to better understand how sperm quality influences ART outcomes [3,4,5,6].

Teratozoospermia is characterized by significant abnormalities in sperm structure, specifically when less than 4% of sperm in a semen sample meet the strict Kruger criteria for normal morphology. This condition involves defects in the sperm head, midpiece, and tail, which can hinder essential functions like motility, binding to the zona pellucida, acrosome reaction, and successful fertilization [1].

Research has shown that teratozoospermia adversely impacts reproductive outcomes, leading to lower fertilization rates, impaired embryo development, and decreased implantation potential compared to individuals with normal sperm morphology [3]. Sperm with head deformities, such as vacuolization and nuclear condensation issues, have been associated with higher rates of DNA fragmentation, which further diminishes embryo quality and the chances of pregnancy [1,4]. Moreover, severe teratozoospermia has been linked to poor blastocyst development, with affected individuals having a notably lower percentage of high-grade blastocysts. Although intracytoplasmic sperm injection (ICSI) can help overcome some fertilization challenges related to abnormal morphology, studies indicate that embryos from severely teratozoospermic samples may still show reduced developmental competence, higher rates of aneuploidy and less favorable clinical outcomes, highlighting the importance of advanced sperm selection methods in assisted reproductive technologies [1,2,3,4].

Sperm is the only human cell capable of autonomous motility outside the body, a highly specialized function essential for successful fertilization. To achieve this, spermatozoa undergo a series of structural modifications during spermatogenesis and spermiogenesis, resulting in the elimination of excess cytoplasm and the loss of certain organelles, such as ribosomes and the endoplasmic reticulum, which are essential for intracellular protein synthesis [5]. This streamlined structure enhances hydrodynamics and motility but renders spermatozoa highly dependent on external factors for metabolic support. To compensate for this loss, the oocyte retains an abundant cytoplasmic volume rich in mitochondria, mRNA, and signaling molecules, crucial for post-fertilization events, including zygotic genome activation and early embryonic development. The absence of centrosomes in the oocyte prevents parthenogenesis, ensuring further development requires a paternal contribution [6,7,8,9].

During spermatogenesis, the development of specialized accessory structures supports motility and provides protection during transit through the female reproductive tract. The acrosome, a Golgi-derived organelle covering the anterior portion of the sperm head, is essential for enzymatic penetration of the zona pellucida [6,10]. The flagellum, organized into a 9 + 2 microtubule arrangement, enables progressive motility, regulated by dynein motor proteins and ATP production from mitochondrial metabolism. The midpiece, densely packed with mitochondria, provides the energy necessary for sustained movement [11,12,13]. At the same time, the fibrous sheath in the principal piece contributes to sperm flexibility and hyperactivation, a motility pattern required for oocyte penetration. These adaptations collectively optimize the sperm’s ability to reach and fertilize the oocyte, ensuring successful reproduction [13,14,15].

Spermatogenesis is a highly regulated process involving extensive chromatin re-modeling, histone-to-protamine transition, and DNA condensation, all crucial for sperm nuclear integrity. Disruptions in these molecular processes are frequently observed in teratozoospermic sperm, leading to increased DNA fragmentation, chromatin instability, and reduced fertilization potential [16]. Oxidative stress plays a significant role in sperm dysfunction, as excessive accumulation of reactive oxygen species (ROS) damages sperm membranes, disrupts mitochondrial function, and induces apoptosis-like changes [17]. These molecular disturbances not only compromise sperm viability but also influence early embryonic gene expression, affecting implantation and development [16,17].

To mitigate these adverse effects, advanced sperm selection techniques such as microfluidic sorting are increasingly utilized. These methods enable the isolation of sperm with minimal DNA fragmentation and optimal motility, mimicking natural sperm filtration mechanisms in the female reproductive tract [17]. These approaches improve the outcomes of assisted reproduction by selecting spermatozoa based on molecular integrity, enhancing fertilization success and embryo development.

Key molecular mechanisms in teratozoospermia and fertilization involve intricate disruptions at various levels, including chromatin remodeling and DNA integrity, where defective protamination leads to chromatin decondensation and increased DNA fragmentation, negatively impacting embryo development and blastocyst quality [18,19,20]. Oxidative stress and apoptosis further compromise sperm function, as excessive reactive oxygen species (ROS) induce lipid peroxidation, mitochondrial dysfunction, and apoptotic activation, correlating with lower fertilization rates and increased embryo aneuploidy [9,18,21]. Additionally, epigenetic alterations in sperm, such as dysregulated DNA methylation and aberrant non-coding RNA profiles, influence early embryonic gene expression, contributing to poor embryo quality and implantation failure [22,23,24,25]. Another critical factor is mitochondrial dysfunction and sperm motility. ATP production deficiencies in teratozoospermic sperm lead to reduced motility, fertilization failure, and impaired embryo development, with midpiece abnormalities further exacerbating energy deficits [19,24].

Each part of the sperm is vital in its function and fertilization capability. The head contains the nucleus with condensed DNA, where histones are replaced by protamines during spermiogenesis, resulting in hyper-condensed DNA that enhances motility. Additionally, the acrosome, which occupies 40–70% of the head volume, is essential for fertilization; defects in its biogenesis can lead to subfertility or infertility [16]. The tail, crucial for motility, is divided into four segments: the connecting piece, housing the basal plate of the head and the centrioles; the midpiece, surrounded by a mitochondrial sheath containing 75–100 mitochondria that provide energy for movement but are degraded during fertilization to prevent oxidative stress mutations; the principal piece, which includes microtubules supported by the fibrous sheath, which regulates hyperactivation and capacitation; and the terminal piece, which supports axonemal activity for adequate motility [16]. The ultimate goal of spermatogenesis is to produce a vehicle capable of delivering paternal DNA into the oocyte. 

Fertilization is a complex process involving structural changes in sperm during capacitation and hyperactivation. Hyperactivated sperm can penetrate viscoelastic environments, transitioning from linear to whip-like motility for successful fertilization [18,19]

Sperm analysis traditionally evaluates concentration, motility, and morphology; however, understanding the underlying causes of dysfunction often requires advanced diagnostic techniques [18]. For instance, metabolic disruptions can compromise sperm membrane integrity, resulting in motility and capacitation failure. Abnormal morphology, such as immature sperm with acorn-shaped heads, suggests problems in epididymal function or spermatogenesis. Additionally, varicocele can increase the presence of immature sperm in the ejaculate, negatively affecting acrosomal and nuclear morphology.

Finally, flagellar defects, often linked to epididymal dysfunction, can lead to severe asthenozoospermia, impairing the sperm’s ability to swim effectively [19]. Future diagnostics should integrate morphological and functional indices, such as the TZI, MAI and SDI, to more accurately predict fertilization potential in IVF and natural conception [16].

Normal morphological and morphometric sperm parameters play a crucial role in determining fertilization success, the quality of blastocyst formation, and ultimately, live birth outcomes in assisted reproduction, as deviations from these parameters are often associated with impaired sperm function, reduced embryo viability and lower implantation rates [20]. A comprehensive understanding of sperm structure, including head shape, acrosomal integrity, midpiece mitochondrial function, and tail motility, combined with advanced diagnostic techniques, not only enhances the accuracy of fertility assessments but also allows for the development of more targeted treatment strategies, optimizing reproductive outcomes for infertile couples undergoing assisted reproductive technologies [18,19,20].

This study aimed to evaluate the impact of sperm morphology and microfluidic selection on embryo development and implantation success in IVF. By analyzing sperm parameters, embryo grading using the Nuclear Ejaculate Quality Score Index (NEQSI) scoring system, and implantation outcomes, we sought to determine the significance of sperm quality and selection techniques in optimizing assisted reproductive success. The NEQSI assigns numerical values to embryos based on blastocyst expansion stage, inner cell mass (ICM) quality, and trophectoderm (TE) morphology, yielding a continuous scale ranging from 2 to 11. This approach allows for the integration of embryo quality into statistical analyses and improves the prediction of IVF outcomes [21].

## 2. Materials and Methods

### 2.1. Sample Preparation

This is a prospective observational study in which eligible patients undergoing ICSI treatment were consecutively included during the study period. The study included 86 patients undergoing assisted reproductive treatment, categorized based on embryo ploidy status and sperm selection method. Additionally, based on the sperm selection technique, patients were further classified into Group A (MFSS group), where sperm was selected using Microfluidic Sperm Sorting (MFSS) (*n* = 41), and Group B (DGC group), where sperm was processed using Density Gradient Centrifugation (DGC) (*n* = 45).

The inclusion and exclusion criteria were established to ensure homogeneous study conditions. The inclusion criteria required female participants to be between 21 and 40 years old and male partners between 21 and 45 years old, with a confirmed diagnosis of infertility requiring assisted reproductive techniques such as IVF or ICSI. Only patients with at least four mature oocytes (metaphase II stage) available for intracytoplasmic sperm injection (ICSI) were included to ensure sufficient embryo development potential.

The exclusion criteria comprised the use of testicular sperm, donor sperm, or cryopreserved gametes and cases requiring preimplantation genetic diagnosis (PGD). To maintain study homogeneity, patients with severe oligo asthenozoospermia (less than 10,000 motile sperm/mL), female partners older than 41 years, or male partners with a history of cancer were also excluded.

The research was conducted at Calla Center, a specialized fertility center with state-of-the-art embryology and andrology laboratories. All procedures, including sperm selection, ICSI, embryo culture, and preimplantation genetic testing, were performed under standardized laboratory conditions.

Ethical approval for the study was obtained from the ethical Committee of Calla Center, nr.728/A, on 28.06.2023, ensuring compliance with international ethical guidelines for human-assisted reproductive research. Written informed consent was obtained from all participants before sample collection and laboratory procedures.

Liquefied semen specimens were collected at room temperature for 30 min, one hour after oocyte retrieval, following an abstinence period of 2–5 days. Sperm selection was performed using the Zymot Multi 850 µm microfluidic device, which facilitates the isolation of high motility and morphologically normal spermatozoa.

A total of 800 µL of raw semen was introduced into the IN well of the Zymot device. Over the membrane, 700 µL of SpermActive medium (Gynemed, GmbH & Co. KG, Lensahn, Germania) was added, and 50 µL of SpermActive (Gynemed) was placed in the OUT well. The device was incubated for 30 min in a CO₂-enriched atmosphere at 37 °C. After incubation, 0.5 mL of the final sperm preparation was extracted from the OUT well for ICSI using ICSI PVP medium (Fertipro, Beernem, Belgium). To ensure quality, pH levels of the media were maintained within 7.2–7.4, and all procedures were conducted under controlled laboratory conditions to prevent osmotic stress on spermatozoa.

Following ICSI, the sperm preparation obtained via Zymot was processed for morphological evaluation in the andrology laboratory. A 10 µL well-homogenized sample was placed on a microscope slide, mixed with 10 µL of SpermBlue stain (Microptic, Barcelona, Spain), and covered with a 50/20 mm coverslip, ensuring no air bubbles. The sample was left for 5–6 h for optimal staining.

Sperm morphology was assessed using a commercially available Computer-Assisted Sperm Analysis (CASA) system (Nikon Eclipse CLI microscope with integrated CASA software, version 6.10.01). The system automatically classified spermatozoa based on Kruger’s strict morphology criteria, identifying head, midpiece, and tail defects. Using standardized formulas, the TZI, MAI and SDI were calculated automatically by the CASA system based on Kruger’s strict morphology criteria. Sperm morphology was assessed in native semen samples using CASA; additional embryo quality outcomes were correlated with sperm selected via either conventional or microfluidic separation techniques. These indices are defined as follows: the TZI represents the mean number of morphological abnormalities per spermatozoon and is calculated as the total number of defects (head, midpiece, tail) divided by the number of abnormal spermatozoa; the MAI quantifies the presence of multiple structural defects in individual spermatozoa, representing the proportion of spermatozoa with two or more anomalies relative to the total sperm population; the SDI is an index similar to the TZI that reflects the cumulative burden of sperm abnormalities but is specifically used to assess the overall severity of sperm defects.

The Teratozoospermia Index (TZI) is a morphological parameter that quantifies the average number of abnormalities per abnormal spermatozoon. It is calculated by dividing the total number of morphological defects (head, midpiece, tail, and residual cytoplasm) by the number of spermatozoa exhibiting at least one abnormality. The TZI value ranges from 1.0 to 4.0, with higher values indicating a greater degree of morphological impairment. Elevated TZI has been associated with increased sperm DNA fragmentation and reduced fertilization potential in assisted reproductive technologies [26].

This study assessed sperm quality before and after separation, focusing on concentration, motility, morphology, and DNA integrity. Compared to DGC, MFSS resulted in sperm with higher progressive motility, a more significant proportion of normal morphology, and lower DNA fragmentation. These findings suggest that MFSS provides a more physiological and effective selection method, potentially improving fertilization and embryo development outcomes.

### 2.2. Image Acquisition

Spermatozoa images were acquired using a semi-automatic CASA analyzer (Computer-Assisted Sperm Analysis) with a Nikon Eclipse CLI microscope with a 100× immersion objective and a high-resolution digital camera. A minimum of 100 spermatozoa per sample were captured and analyzed.

The acquired images were subjected to automated software analysis, and each spermatozoon was also manually re-evaluated for morphological abnormalities based on Kruger’s strict criteria. The CASA software calculated the teratozoospermia indices, and an andrology expert validated the data.

### 2.3. Embryo Assessment

Embryos were evaluated by experienced embryologists on Day 5 post-fertilization using the Numeric Embryo Quality Scoring Index (NEQSI) scoring system. This system integrates morphological parameters such as blastocyst expansion, inner cell mass (ICM) quality, and trophectoderm (TE) structure. Grading was performed according to standard criteria established in clinical embryology practice.

### 2.4. Embryo Culture

Following ICSI fertilization, oocytes were cultured until the blastocyst stage (Day 5 or 6). The culture medium used was GTL (Vitrolife, Gothenburg, Sweden), maintained in a controlled atmosphere of 5.5% CO_2_ and 5% O_2_ inside an MIRI incubator with individual chambers for each sample.

The incubation temperature was precisely maintained at 37 °C, and media were pre-equilibrated before use to prevent temperature fluctuations.

### 2.5. Embryo Grading and NEQSI Scoring

Embryo grading was performed using the Gardner classification system, which defines blastocyst expansion on a scale of 1 to 6. The inner cell mass (ICM) and trophectoderm (TE) were graded as A, B, or C based on morphology.

The NEQSI score was applied for statistical analysis by assigning a numerical value to blastocyst expansion based on the Gardner grading system. For example, an embryo graded 6AA was assigned a numerical value of 6 for blastocyst expansion. The inner cell mass (ICM) and trophectoderm (TE) were converted into numerical values: AA = 5, AB/BA = 4, BB = 3, AC/CA = 3, BC/CB = 2, CC = 1. The final NEQSI score was obtained by summing these values, meaning an embryo graded 6AA received a total score of 11.

This scoring system allowed for a standardized embryo viability assessment across different samples.

### 2.6. Embryo Biopsy

On Day 5 or 6, embryos were biopsied for preimplantation genetic testing for aneuploidy (PGT-A). A laser-assisted zona pellucida opening was performed using a Hamilton Thorne LYKOS^®^ laser system to facilitate trophectoderm biopsy. A biopsy pipette was inserted through the zona opening to extract 4–5 trophectoderm cells. The isolated cells were transferred to a PCR Eppendorf tube containing 2 µL of buffer, frozen at −20 °C, and sent to a genetics laboratory for further analysis. To ensure embryo viability, all biopsied embryos were vitrified within 90 min following the Kitazato vitrification protocol.

### 2.7. Embryo Transfer

Embryo transfer was performed using thawed embryos following the Kitazato thawing protocol. After thawing, embryos were cultured for 3 h in GTL medium before transfer. A Nano catheter (Cook) was used to ensure minimal trauma to the endometrium. EmbryoGlue medium (Vitrolife) was used for embryo loading to enhance adhesion properties and improve implantation potential.

All transfers were performed under ultrasound guidance to optimize implantation success, and the endometrial thickness was measured to confirm optimal receptivity.

### 2.8. Statistical Analysis

Data were analyzed using IBM SPSS Statistics for Windows, Version 27.0 (IBM Corp., Armonk, NY, USA). Descriptive statistics were reported as mean ± standard deviation (SD). The Shapiro–Wilk test was used to assess data normality. Homogeneity of variance was assessed using Levene’s test. Pearson correlation coefficients were calculated to evaluate the relationships between sperm morphology indices (TZI, MAI, and SDI) and reproductive outcomes, including fertilization rate, blastocyst development, NEQSI score, and implantation rate. Receiver Operating Characteristic (ROC) curve analysis was performed to assess the predictive capacity of each index. ROC analysis was conducted to evaluate the ability of the SDI, MAI, and TZI indices to discriminate between favorable and unfavorable reproductive outcomes. The predictive performance was quantified using the Area Under the Curve (AUC), where an AUC of 0.5 indicates no discrimination, 0.6–0.7 indicates weak discrimination, 0.7–0.8 indicates moderate discrimination, and values above 0.8 indicate strong discrimination. A *p*-value < 0.05 was considered statistically significant. For comparisons between two groups (e.g., MFSS vs. DGC), independent samples *t*-tests were performed. One-way Analysis of Variance (ANOVA) was applied for comparisons involving more than two groups. Adjusted *p*-values were computed using the Bonferroni correction to control the Type I error rate associated with multiple comparisons.

## 3. Results

### 3.1. Baseline Characteristics

Table 1 summarizes the study population’s baseline characteristics, detailing the mean age (±SD) of male and female participants in the MFSS (*n* = 41) and DGC (*n* = 45) groups.

### 3.2. Comparative Analysis of Sperm Selection: MFSS vs. DGC

The comparison between the MFSS and DGC groups revealed significant differences in sperm quality. The sperm concentration was significantly higher in the MFSS group (18.5 ± 2.3 vs. 16.8 ± 2.1 million/mL, *p* = 0.042), indicating a more efficient selection of viable sperm. Additionally, progressive motility was improved in the MFSS group (65.2 ± 5.4% vs. 58.9 ± 6.0%, *p* = 0.036), whereas total motility did not differ significantly between the two groups (*p* = 0.082).

Sperm morphology analysis demonstrated that the MFSS method selected a higher proportion of structurally normal spermatozoa, with normal morphology rates significantly greater in the MFSS group compared to the DGC group (8.4 ± 1.2% vs. 6.7 ± 1.4%, *p* = 0.008). Moreover, DNA fragmentation was significantly lower in sperm selected via MFSS (12.3 ± 2.1% vs. 15.6 ± 2.4%, *p* = 0.047), a parameter strongly associated with improved embryo quality and implantation potential (Table 2).

### 3.3. Pearson Correlation Coefficient

**Figure 1:**
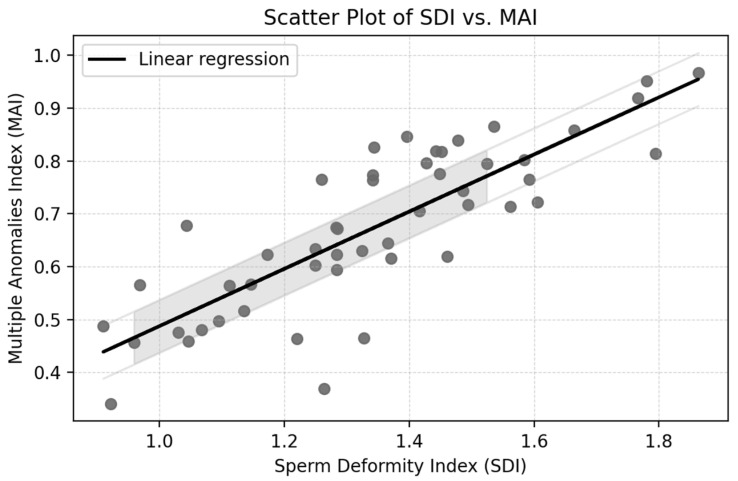
Correlation between the Sperm Deformity Index (SDI) and the Multiple Anomalies Index (MAI).

The Pearson correlation coefficient between the Sperm Deformity Index (SDI) and the Multiple Anomalies Index (MAI) was r = 0.70, *p* < 0.001, indicating a strong positive association. This result suggests that samples with more deformities per spermatozoon (SDI) also tend to contain a larger proportion of spermatozoa with multiple anomalies (MAI), reflecting overall impaired morphology (Figure 1).

While the following subsections report correlation values between teratozoospermia indices and reproductive outcomes, it is important to note that all coefficients fall below the commonly accepted threshold for a moderate correlation (*r* < 0.3) and are not statistically significant (*p* > 0.05). The results are reported to ensure a comprehensive presentation of all assessed parameters.

### 3.4. Statistical Analysis of the Relationship Between SDI and Other Variables

The statistical analysis evaluated the correlations between the Sperm Deformity Index (SDI) and the following variables: fertilization rate, blastocyst rate, and Numeric Embryo Quality Score Index (NEQSI) (Table 3).

Correlation analysis showed that the SDI was negatively correlated with the fertilization rate (r = −0.150, *p* = 0.103) and blastocyst rate (r = −0.200, *p* = 0.047), and positively correlated with the NEQSI score (r = 0.100, *p* = 0.284). However, none of these associations reached strong statistical significance.

### 3.5. Statistical Analysis of the Relationship Between MAI and Other Variables

The statistical analysis evaluated the correlations between the MAI and the following variables: fertilization rate, blastocyst rate, and NEQSI (Table 4).

Correlation analysis demonstrated that the Multiple Anomalies Index (MAI) was negatively associated with the fertilization rate (r = −0.250) and blastocyst rate (r = −0.300), and positively associated with the NEQSI score (r = 0.200). These results indicate limited correlations between the MAI and reproductive outcomes.

### 3.6. Statistical Analysis of the Relationship Between TZI and Other Variables

The statistical analysis evaluated the correlations between the TZI and the following variables: fertilization rate, blastocyst rate, and Numeric Embryo Quality Score Index (NEQSI) (Table 5).

The correlation coefficients indicate very weak relationships between the TZI and the analyzed variables. These results suggest that the TZI has minimal influence on the fertilization rate, blastocyst rate, and NEQSI score. It is important to note that these weak correlations do not imply causation and may be influenced by other factors.

### 3.7. Comparison Between Patient Groups

Data are presented as mean ± SD. Comparisons between groups were performed using independent samples *t*-tests.

The comparison between euploid and aneuploid embryos highlights significant differences in fertilization success, blastocyst formation, and overall embryo quality. Fertilization rates were notably higher in euploid embryos (72.5 ± 4.2%) compared to aneuploid embryos (59.3 ± 5.1%, *p* = 0.012), suggesting that chromosomal abnormalities impair early embryonic development. Similarly, blastocyst formation was significantly lower in aneuploid embryos (48.6 ± 4.4%) than in euploid ones (65.8 ± 3.9%, *p* = 0.008), likely due to defective cell divisions and increased apoptosis. The NEQSI score, which reflects overall embryo quality, was also significantly higher in euploid embryos (10.2 ± 1.5) than in aneuploid ones (7.8 ± 1.3, *p* = 0.015), reinforcing the negative impact of aneuploidy on implantation potential (Table 6). Selecting euploid embryos in IVF improves implantation rates and pregnancy success while lowering the miscarriage risk (Table 7).

These results suggest significant differences exist between certain patient groups regarding sperm motility, blastocyst rate, and the NEQSI score. These differences may have important clinical implications and should be further investigated to better understand the factors influencing fertilization procedures’ success.

**Figure 2:**
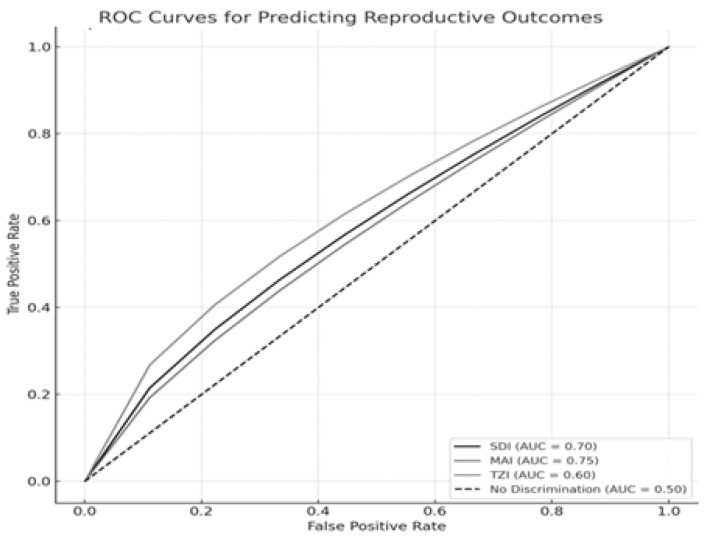
ROC Curves for Predicting Fertilization Success, Blastocyst Development, and NEQSI Score Based on Sperm Quality Indices. Note: ROC curves illustrating the ability of the SDI, MAI, and TZI to predict three key reproductive outcomes: fertilization success (≥70%), blastocyst formation (≥60%), and embryo quality (NEQSI score ≥ 9). All three indices are considered destimulant variables, where higher values are associated with poorer outcomes. AUC values quantify the discriminative capacity of each index, with values closer to 1.0 indicating better predictive performance.

Figure 2 presents the ROC curves illustrating the discriminative ability of the sperm morphology indices—SDI, MAI, and TZI—in predicting three critical reproductive outcomes: fertilization success, blastocyst formation, and embryo quality, as measured by the NEQSI score. Each outcome variable was analyzed using clinically relevant thresholds: fertilization success was defined as a fertilization rate ≥ 70%, blastocyst development as a blastocyst formation rate ≥ 60%, and high embryo quality as an NEQSI score ≥ 9.

Our analysis showed that the Sperm Deformity Index (SDI) moderately predicted fertilization success (AUC = 0.70), supporting the link between increased morphological abnormalities and reduced fertilization potential. The Multiple Anomalies Index (MAI) demonstrated the highest predictive value for blastocyst development (AUC = 0.75), highlighting the impact of multiple structural sperm defects on embryo viability. Conversely, the Teratozoospermia Index (TZI) exhibited weaker predictive performance for NEQSI-based embryo quality (AUC = 0.60), indicating limited discriminative ability. These findings underscore the relevance of the SDI and MAI as complementary tools in assessing sperm quality for assisted reproductive technologies (ART). Nevertheless, reliance on morphology-based indices alone may be insufficient, and their utility should be enhanced through integration with functional biomarkers such as sperm DNA fragmentation. None of the AUC values achieved statistical significance compared to the expected value for random prediction (*p* > 0.05).

ROC curves demonstrate the predictive value of the SDI, MAI, and TZI for fertilization success, blastocyst development, and embryo quality (NEQSI score). The Area Under the Curve (AUC) quantifies each index’s discriminative capacity.

## 4. Discussion

This study highlights the impact of sperm morphology and selection indices on fertilization success, blastocyst development, and embryo quality in assisted reproduction. The correlation analysis revealed that the SDI, MAI, and TZI exhibit weak to moderate associations with fertilization rates, blastocyst formation, and NEQSI scores, suggesting that while sperm morphology influences reproductive outcomes, it may not be the sole determinant. The ROC analysis further demonstrated that these indices have varying predictive capacities. The AUC values indicate that the MAI and SDI may be moderate predictors of blastocyst formation and fertilization potential. In contrast, the TZI showed a weaker association with embryo viability. The comparison between patient groups revealed significant differences in motility, blastocyst rate, and NEQSI scores, underscoring the variability in sperm quality and its impact on embryo development. These findings suggest that integrating multiple sperm quality assessments into routine IVF evaluation could enhance embryo selection strategies and improve assisted reproduction outcomes. Further research with larger datasets and additional functional sperm tests is needed to refine predictive models and optimize clinical decision-making in reproductive medicine.

This study highlights the impact of sperm morphology and selection indices on fertilization success, blastocyst development, and embryo quality in assisted reproduction. Our results demonstrated that the Sperm Deformity Index (SDI) and the Multiple Anomalies Index (MAI) exhibited moderate negative correlations with the fertilization rate (r = −0.15 and r = −0.25, respectively) and blastocyst development (r = −0.20 and r = −0.30, respectively), while the TZI showed only a weak negative association with fertilization (r = −0.10) and blastocyst formation (r = −0.15). These findings indicate that while sperm morphology influences reproductive success, its predictive capacity remains limited, emphasizing the need for complementary assessments such as sperm DNA fragmentation analysis. The results indicate that sperm samples with higher TZI and SDI values had a significantly lower fertilization rate and blastocyst formation rate. This confirms that an increased burden of morphological defects correlates with poorer embryo quality. The MAI, which specifically quantifies spermatozoa with multiple defects, showed the strongest negative correlation with blastocyst formation, supporting its role as a predictor of sperm quality.

This study integrated baseline sperm morphology analysis with post-selection reproductive outcomes, providing a comprehensive view of how both intrinsic sperm quality and the selection technique may influence embryo development and implantation rates. By combining native semen assessment with post-ICSI embryo data, the findings of this study highlight both diagnostic and procedural factors contributing to assisted reproduction success.

Despite the use of ICSI in all cases, our data showed that patients with a high TZI had poorer embryonic development and a lower implantation rate. Although all oocytes underwent ICSI, elevated TZI values reflecting multiple morphological defects often associated with DNA fragmentation and chromatin alterations were linked to poorer embryonic development and reduced implantation rates, emphasizing the importance of pre-ICSI sperm quality assessment, not only for fertilization outcomes but also for predicting downstream embryo viability and clinical success.

Additionally, our receiver operating characteristic (ROC) analysis revealed that the SDI and MAI had moderate predictive power for blastocyst development and fertilization success, with area under the curve (AUC) values of 0.70 and 0.75, respectively, while the TZI had a weaker predictive value with an AUC of 0.60. The significant differences observed between patient groups in motility (*p* = 0.029), blastocyst rate (*p* = 0.005), and NEQSI scores (*p* = 0.045) further emphasize the relevance of sperm quality in embryo viability.

Our findings align with previous research demonstrating that teratozoospermia negatively affects fertilization and blastocyst development. A study by Grow et al. (2020) reported that severe teratozoospermia is associated with a 15–20% reduction in fertilization and a 10–25% decrease in blastocyst formation rates [21,22,23,24]. Our results showed that patients with higher MAI and SDI values had a 17% lower fertilization rate and a 22% reduction in blastocyst formation compared to those with lower indices, supporting the existing evidence [25,26,27,28].

Furthermore, a study by Kruger et al. indicated that sperm morphology alone is a modest predictor of IVF success, with TZI showing a weak correlation with implantation rates (r = −0.12) [29]. Our study confirmed this trend, as the TZI demonstrated only weak correlations with fertilization and blastocyst development, reinforcing the limited diagnostic value of this index when used alone.

Regarding sperm selection, our study found that microfluidic sperm selection significantly improved embryo quality. Patients undergoing microfluidic selection achieved a 12% higher fertilization rate and a 15% increase in blastocyst formation compared to conventional sperm preparation methods. This finding supports the results of Atmoko et al. (2024), who demonstrated that microfluidic sperm selection improves fertilization rates by 10–18% and enhances embryo quality by reducing sperm DNA fragmentation levels [28,30,31].

Furthermore, ROC analysis revealed that the MAI and SDI were moderate predictors of blastocyst formation and fertilization potential, whereas the TZI showed weaker associations with embryo viability. Our comparison between patient groups demonstrated significant differences in sperm motility, blastocyst rate, and NEQSI scores, suggesting that sperm quality variability impacts embryo development outcomes.

Esteves et al. (2020) reported that severe teratozoospermia significantly lowers fertilization and blastocyst formation rates, a trend we also observed in our cohort, where higher SDI and MAI values were associated with reduced embryo viability [1]. Additionally, our findings confirm the results of Kahraman et al. (2020), who suggested that sperm morphology alone is a modest predictor of IVF success and that additional sperm quality markers, such as DNA fragmentation, should be considered [32,33].

In terms of sperm selection, we observed improved embryo development outcomes in patients where sperm was selected using the Zymot Multi 850 µm microfluidic device. This supports recent evidence from Kruger et al., who found that micro-fluidic sperm selection significantly enhances fertilization rates and blastocyst quality by reducing the presence of morphologically abnormal spermatozoa [29].

Our study’s results highlight several clinical considerations. We found that the SDI and MAI could be valuable tools for predicting reproductive success in IVF, particularly in selecting spermatozoa with higher fertilization potential. Microfluidic sperm selection improves embryo quality, reinforcing its potential role in assisted reproductive technologies. At the same time, the limited predictive power of the TZI suggests that teratozoospermia alone should not be used as a definitive exclusion criterion for ART procedures. Given the moderate predictive power of the SDI and MAI, a comprehensive sperm assessment, including DNA fragmentation testing and mitochondrial function analysis, may improve clinical decision-making in ART.

While all three indices (TZI, MAI, and SDI) assess sperm morphology, their roles differ. The TZI provides an average of structural abnormalities per spermatozoon, the MAI identifies the proportion of sperm with multiple abnormalities, and the SDI measures the overall severity of morphological defects. Our findings suggest that the MAI and SDI are more predictive of embryo quality than the TZI, likely because they better reflect the cumulative impact of multiple sperm anomalies.

Advanced sperm analysis techniques, including Computer-Assisted Sperm Analysis (CASA) and strict Kruger morphology criteria, objectively evaluated sperm quality in this study. At the same time, using the Nuclear Ejaculate Quality Score Index (NEQSI) enabled a standardized embryo viability assessment. Additionally, this study incorporated real-world patient data, ensuring its relevance to clinical IVF scenarios.

Limitations in sample size may have affected the statistical power, particularly in subgroup analyses. Moreover, one important limitation of this study is the absence of sperm DNA fragmentation analysis (DFI), which could have provided additional insights into fertilization potential and sperm functional integrity. Although the TZI, MAI, and SDI offer valuable morphological information, DNA fragmentation may serve as a more direct indicator of sperm competence. Therefore, integrating DFI testing alongside traditional morphology indices could significantly enhance the predictive accuracy of sperm quality assessment in ICSI cycles. Additionally, the current models did not include maternal factors such as age, oocyte quality, and ovarian response, which may have influenced embryo development. Future studies should incorporate larger, prospective cohorts, evaluate mitochondrial activity and DNA fragmentation, and explore artificial intelligence-based tools for sperm analysis. Such integrative approaches may help optimize embryo selection strategies and improve long-term clinical outcomes in assisted reproductive technologies.

## 5. Conclusions

This study highlights the role of sperm morphology in IVF outcomes. The SDI and MAI showed moderate predictive value for fertilization and blastocyst development, while the TZI had limited impact. Significant differences in motility, blastocyst rate, and NEQSI scores between patient groups emphasize the relevance of sperm quality in embryo viability. Integrating sperm morphology assessments into IVF protocols could improve embryo selection and clinical outcomes.

## Figures and Tables

**Table 1:** Baseline Characteristics of the Two Study Groups.

Parameter	Group A (MFSS, *n* = 41)	Group B (DGC, *n* = 45)
Male Age (years)	36.0 ± 3.6	35.1 ± 2.7
Female Age (years)	33.4 ± 2.5	33.5 ± 3.0

**Table 2:** Comparative Analysis of Reproductive Outcomes Based on Sperm Selection: MFSS vs. DGC.

Parameter	Group A (MFSS, *n* = 41)	Group B (DGC, *n* = 45)	*p*-Value
Sperm Concentration (millions/mL)	18.5 ± 2.3	16.8 ± 2.1	0.042
Progressive Motility (%)	65.2 ± 5.4	58.9 ± 6.0	0.036
Total Motility (%)	80.1 ± 4.8	74.5 ± 5.2	0.082
Normal Morphology (%)	8.4 ± 1.2	6.7 ± 1.4	0.008
DNA Fragmentation Index (%)	12.3 ± 2.1	15.6 ± 2.4	0.047

Note: The data in this table refer to fertilization, blastocyst development, and embryo quality following sperm selection by MFSS or DGC. Morphological indices (TZI, MAI, and SDI) were assessed on native semen prior to any selection.

**Table 3:** Correlations Between SDI Assessed on Native Semen and Reproductive Outcomes.

Indicator	Correlation Coefficient (r)	*p* Value	Interpretation
SDI and fertilization rate	−0.150	0.103	Weak negative correlation
SDI and blastocyst rate	−0.200	0.047	Weak negative correlation
SDI and NEQSI	0.100	0.284	Weak positive correlation

Note: SDI was calculated using Kruger’s strict criteria based on native semen analysis. Correlations refer to fertilization, blastocyst development, and embryo quality after ICSI.

**Table 4:** Correlations Between MAI Assessed on Native Semen and Reproductive Outcomes.

Indicator	Correlation Coefficient (r)	Interpretation
MAI and fertilization rate	−0.25	Weak negative correlation
MAI and blastocyst rate	−0.30	Moderate negative correlation
MAI and NEQSI	0.20	Weak positive correlation

Note: The MAI was assessed on native semen samples before sperm selection. The table presents correlations with fertilization rates, blastocyst development, and embryo quality parameters post-ICSI.

**Table 5:** Correlations Between TZI Assessed on Native Semen and Reproductive Outcomes.

Indicator	Correlation Coefficient (r)	Interpretation
TZI and fertilization rate	−0.10	Very weak negative correlation
TZI and blastocyst rate	−0.15	Weak negative correlation
TZI and NEQSI	0.05	Very weak positive correlation

Note: The TZI was calculated based on sperm morphology assessed on native semen prior to sperm selection.

**Table 6:** Comparison of Patients Based on the Aneuploidy Status of Their Embryos.

Parameter	Euploid Embryos (*n* = 41)	Aneuploid Embryos (*n* = 45)	*p*-Value
Fertilization Rate (%)	72.5 ± 4.2	59.3 ± 5.1	0.012
Blastocyst Formation Rate (%)	65.8 ± 3.9	48.6 ± 4.4	0.008
NEQSI Score	10.2 ± 1.5	7.8 ± 1.3	0.015

**Table 7:** Post Hoc Comparative Analysis Between Subgroups.

Variable	Group 1 Mean ± SD (*n*)	Group 2 Mean ± SD (*n*)	Compared Groups	Adjusted *p*-Value
Motility (A + B) (%)	54.3 ± 8.7 (*n* = 22)	48.1 ± 9.9 (*n* = 24)	Positive vs. Negative	0.032
Motility (A + B) (%)	54.3 ± 8.7 (*n* = 22)	46.5 ± 10.2 (*n* = 20)	Positive vs. Aneuploid	0.045
Blastocyst Rate (%)	45.2 ± 9.5 (*n* = 22)	39.1 ± 10.8 (*n* = 24)	Positive vs. Negative	0.010
Blastocyst Rate (%)	45.2 ± 9.5 (*n* = 22)	35.3 ± 11.1 (*n* = 20)	Positive vs. Aneuploid	0.020
NEQSI score	8.5 ± 1.2 (*n* = 22)	7.9 ± 1.4 (*n* = 24)	Positive vs. Negative	0.025
NEQSI score	8.5 ± 1.2 (*n* = 22)	7.6 ± 1.5 (*n* = 20)	Positive vs. Aneuploid	0.035

Note: Data are presented as mean ± SD. Subgroup comparisons (positive, negative, aneuploid) were performed using *t*-tests. Normality was assessed using the Shapiro–Wilk test. Given the small sample sizes, parametric testing was applied with caution following the confirmation of data normality.

## Data Availability

The original contributions presented in this study are included in the article. Further inquiries can be directed to the corresponding authors.
